# Murine Thigh Microdialysis to Evaluate the Pharmacokinetic/Pharmacodynamic Integration of Cefquinome Against *Actinobacillus pleuropneumoniae*

**DOI:** 10.3389/fvets.2020.00448

**Published:** 2020-08-05

**Authors:** Longfei Zhang, Zichong Zhou, Xiaoyan Gu, Sixiu Huang, Xiangguang Shen, Huanzhong Ding

**Affiliations:** ^1^College of Animal Science and Veterinary Medicine of Henan Institute of Science and Technology, Xinxiang, China; ^2^Guangdong Provincial Key Laboratory of Veterinary Drugs Development and Safety Evaluation, South China Agricultural University, Guangzhou, China

**Keywords:** microdialysis, cefquinome, PK/PD integration, murine thigh infection model, *Actinobacillus pleuropneumoniae*

## Abstract

This study aimed to explore the application of microdialysis in pharmacokinetic (PK)/pharmacodynamic (PD) integration of cefquinome against *Actinobacillus pleuropneumoniae*. After the *A. pleuropneumoniae* population reached 10^6^ CFU/thigh, the mice received 0.04, 0.16, 0.63, 2.5, and 10 mg/kg cefquinome by subcutaneous injection. Plasma samples were collected by retro-orbital puncture for 4 h, and thigh dialysate was obtained by microdialysis at a flow rate of 1.5 μL/min for 6 h for the PK study. For the PD experiment, the infected mice were treated with a 4-fold-increase in the total cefquinome dose, ranging from 0.01 to 10 mg/kg/24 h, divided into one, two, three, four, and eight doses. The number of bacteria was determined and an inhibitory sigmoid maximum effect (E_max_) model was used to analyse the relationships between PK/PD parameters and efficacy. The mean penetration of cefquinome from plasma to the thigh was 0.591. The PK data for PK/PD integration were obtained by extrapolation. The fittest PK/PD parameter for efficacy evaluation was %*f*T>MIC (the percentage of time that free drug concentrations exceed the MIC). The magnitudes of %*f*T>MIC to achieve net bacterial stasis, 1-log_10_ CFU reduction, 2-log_10_ CFU reduction, and 3-log_10_ CFU reduction were 19.56, 28.65, 41.59, and 67.07 % in plasma and 21.74, 36.11, 52.96, and 82.68% in murine thigh, respectively. Microdialysis was first applied to evaluate the PK/PD integration of cefquinome against *A. pleuropneumoniae*. These results would provide valuable references when we apply microdialysis to study the PK/PD integration model and use cefquinome to treat animal diseases caused by *A. pleuropneumoniae*.

## Introduction

Cefquinome is a fourth-generation cephalosporin that is used solely for treating veterinary diseases. It has been approved for the treatment of respiratory tract disease caused by *Pasteurella multocida, Streptococcus pneumoniae, Klebsiella pneumoniae*, and *Actinobacillus pleuropneumoniae* in pigs and calves (1). Because of the wide application of antibacterial drugs, bacteria have gradually developed resistance (2–4). Inappropriate dosage regimens is a common cause of bacterial resistance. To optimize dosage schedules and prolong usage of antibacterial drugs, the pharmacokinetics of drugs, bacteria-killing mechanisms, and antibacterial efficacy in target species have been studied in many experiments. One of the common methods for designing a rational dosing regimen is a pharmacokinetic (PK) and pharmacodynamic (PD) integration model (5–7). Further, an *in vivo* infection model is an efficient model that can truly reflect dynamic interactions between hosts, pathogens, and antibacterial agents.

To evaluate the relationships between PK/PD parameters and the antibacterial effect *in vivo*, several animal infection models have been applied, such as tissue cage models (8–14), murine lung infection model (15–17), and murine thigh infection model (18–21). However, there are still some deficiencies in these models. For example, the tissue cage infection model is considered to reflect the interactions between drugs, microorganisms, and hosts; however, the results were obtained from tissue cages that were sealed by granulation tissue. Therefore, invaded and destroyed cells and interstitial space were not taken into consideration when a bacterial infection developed in the target tissues. For the lung and thigh infection model, bacterial infection was induced in parenchymal organs but the PK indices of the drugs used were obtained from plasma when correlated the PK/PD parameters to efficacy. Therefore, it is valuable and necessary to explore the combination of free drugs in infected tissue and antibacterial effects when we design rational dosing regimens.

Microdialysis is a microsampling technique that can be used to monitor free drug concentrations in the target tissue with minimal invasion (22). Since it was first developed, microdialysis (23) has been used to study the PK characteristics of several antibacterial drugs, such as tobramycin (24), florfenicol (25), levofloxacin (26), and cefpodoxime (27) in the interstitial fluid of organs. Therefore, it is a suitable method to study the relationships between PK/PD indices of free drugs and the antibacterial effect in target tissues.

In our present study, we applied a microdialysis method to determine the PK parameters of free cefquinome in murine thigh and plasma in neutropenic murine thigh infected with *A. pleuropneumoniae*. We also analyzed the relationships between antibacterial efficacy and PK/PD parameters of free cefquinome derived from the murine thigh. There are three objects of this study. The first aim was to explore the pharmacokinetics of cefquinome in the infected murine thigh and plasma by microdialysis. The second aim was to assess the relationships between efficacy and PK/PD parameters of free cefquinome derived from the murine thigh. The last aim was to evaluate the magnitudes of PK/PD parameters required for achieving the antibacterial effect of cefquinome against *A. pleuropneumoniae*.

## Materials and Methods

### Chemicals and Microdialysis System

The cefquinome standard was purchased from China Institute of Veterinary Drugs Control (Beijing, P. R. China). Pentobarbital sodium was purchased from Jian Yang Biotechnology Co., Ltd. Nicotinamide adenine dinucleotide (NAD, lot: 20160810) was provided by MYM Biological Technology Company Limited. Ringer's solution and cyclophosphamide were provided by Shanghai yuanye Bio-Technology Co., Ltd. The microdialysis system was provided by Bioanalytical System, Inc. (West Lafayette, USA). The components of microdialysis included BASi syringe pumps, a single syringe pump drive, a drive controller (Worker Controller), a gas-tight syringe (1.0 mL), and an MD-2000 linear microdialysis probe (membrane length: 10 mm, cut-off: 30 kDa).

### Bacterial Strain and Minimum Inhibitory Concentration (MIC)

The standard strain of *A. pleuropneumoniae* (CVCC259) was supplied by China Veterinary Culture Collection Center (Qingdao, China). The bacteria were cultivated in Tryptic Soy Broth (TSB) and Mueller-Hinton Agar (MHA) (Guangdong Huankai Microbial Technology) supplemented with new-born bovine serum (4%, V/V; Guangzhou Ruite Biotechnology Ltd) and NAD (10 mg/L).

After incubation for 8 h in TSB in a constant-temperature shaker maintained at 37°C and 200 rpm/min, the logarithmic-phase bacteria were diluted and a final concentration of 5 × 10^5^ colony-forming units (CFU)/mL was applied to test the MIC by the microdilution method (Clinical and Laboratory Standards Institute, CLSI) (28).

### Establishment of the Neutropenic Murine Thigh Infection Model

250 specific-pathogen-free female ICR mice (6-week-old, 25 to 30 g) were obtained from Hunan SJA Laboratory Animal Co., Ltd. (Hunan, China) and housed in Laboratory Animal Center of South China Agricultural University. Water and fodder were available *ad libitum*. All the experimental protocols were approved by the Committee on the Ethics of Animals of South China Agricultural University (Approval number: 2017043).

After acclimatization for 7 days, the mice received cyclophosphamide by intraperitoneal injection at 4 days (150 mg/kg) and 1 day (100 mg/kg) prior to infection. When the mouse model of neutropenia was established, 0.1 mL of suspension with a bacterial population of 10^6^ to 10^7^ CFU/mL was injected into each thigh muscle by intramuscular injection. After allowing growth for 2 h, the mice with a bacterial concentration of 10^6^ CFU/thigh were used in subsequent experiments. During the dialysis procedure, the mice were placed in a special device which could fix head and exposure thigh. And then, the murine thigh were restrained for dialysis study.

### Calibration of Microdialysis

The *in vitro* relative recovery (RR) was determined by dialysis and retrodialysis (29). Different concentrations of cefquinome (50, 100, and 500 ng/mL) and flow rates (0.5, 1.0, 1.5, and 2.0 μL/min) were studied. The perfusate applied was Ringer's solution. The formulas to calculate the RRs by dialysis (RR_dialysis_) and retrodialysis (RR_retrodialysis_) were as follows:

(1) $$\begin{array}{r} {\text{RR}}_{\text{dialysis}}\left(\%\right)=\left({\text{C}}_{\text{dial}}/{\text{C}}_{\text{ext}}\right)\times 100\% \end{array}$$

(2) $$\begin{array}{r} {\text{RR}}_{\text{retrodialysis}}\left(\%\right)=\left(\left({\text{C}}_{\text{perf}}-{\text{C}}_{\text{dial}}\right)/{\text{C}}_{\text{perf}}\right)\times 100\% \end{array}$$

where C_dial_ is the drug concentration in the dialysate, C_ext_ is the drug concentration in the Ringer's solution around the microdialysis probe, and C_perf_ is the drug concentration in the perfusate.

The *in vivo* RR was determined by retrodialysis and the microdialysis probe was implanted into the thigh muscle when the mice were anesthetized with 1.5% pentobarbital sodium at 0.005 mL/g of body weight by intraperitoneal injection. Briefly, the MD-2000 linear probe was implanted (1 h later after infection) in the murine thigh by an introducer needle (25G needle). After the dialysis membrane was entirely surrounded by the thigh muscle, the inlet and outlet tubes of the probe were fixed to maintain the dialysis membrane immobile. Thereafter, the inlet of the probe was connected to the syringe pump drive and perfused with blank perfusate at a flow rate of 1.5 μL/min to stabilize the microdialysis system until the mice recovered consciousness. The *in vivo* RR was measured using perfused Ringer's solution with 500 ng/mL cefquinome at 1.5 μL/min and collected dialysate samples every 15 min for 6 h.

To determine the plasma protein binding of cefquinome, the blank plasma samples were obtained from infected mice and detected by microdialysis as described by Bernardi (24). Different concentrations of cefquinome (5, 50, and 500 ng/mL) were explored.

### Pharmacokinetics of Free Cefquinome in Murine Plasma and Thigh

After the murine thigh infection model was established, the mice received single doses of cefquinome (2 h after the mice received bacterial suspension) at 0.04, 0.16, 0.63, 2.5, and 10 mg/kg of body weight by subcutaneous injection. One hundred microliter blood was collected in heparin-containing tubes at 0.08, 0.17, 0.25, 0.5, 1, 2, 3, and 4 h by retro-orbital puncture (24 mice/each experiment). After centrifugation at 3000 × *g* for 10 min, the supernatant was transferred into cryotubes and stored at −20°C until analysis within 2 weeks. The thigh dialysate was obtained by microdialysis every 15 min at a flow rate of 1.5 μL/min for 6 h and stored at −20°C until analysis within 2 weeks. The mice were sacrificed by CO_2_ asphyxiation at the end of the study. The plasma and thigh dialysate samples collected at every time point were repeated eight times for every dosage (8 mice/each experiment).

Both the plasma and dialysate samples were detected using an Agilent 1,200 series high-performance liquid chromatography (HPLC) unit and an Agilent 6,410 triple quadrupole mass spectrometer equipped with an electrospray ionization source (HPLC–MS/MS, Agilent Technologies). Before the plasma samples were analyzed, isopycnic acetonitrile was added to plasma and centrifuged at 12,000 × *g* for 10 min. The supernatant was mixed with ultrapure water (1:4, V/V) and filtered through a 0.22-μm nylon syringe filter (Jin Teng Experiment Equipment Company). For calibration, the standard curve (*R*^2^ > 0.99) was defined by six calibration standards of cefquinome in plasma, ranging from 5 to 200 ng/mL. For the dialysate, 20 μL of the sample was mixed with 80 μL of ultrapure water and then transferred to an autosampler vial for detection. The standard curve (*R*^2^ > 0.99) was determined using eight calibration standards of cefquinome in the dialysate, ranging from 2.5 to 500 ng/mL. The lower limit of quantification was 5 ng/mL and 2.5 ng/mL for plasma and thigh dialysate, respectively.

A non-compartmental model was used to analyze the PK parameters of free cefquinome in murine plasma and thigh by using WinNonlin software (version 5.2; Pharsight, MO, USA). The PK parameters include the elimination half-life (T_1/2β_), the area under the concentration-time curve (AUC), and the maximum drug concentration (C_max_).

### PK/PD Integration of Cefquinome Against *A. pleuropneumoniae*

For PD analysis, the infected mice were treated with cefquinome at six total doses (0.01, 0.04, 0.16, 0.63, 2.5, and 10 mg/kg), which were divided by five dosing intervals (every 3, 6, 8, 12, and 24 h) over the 24-h period by subcutaneous injection. Groups of two mice were used for each dosing regimen. The mice were sacrificed and the thighs were immediately removed for bacterial determination 24 h after drug administration. The murine thighs were homogenized in 10 mL of 0.9% sterile physiological saline solution and diluted by 10-fold dilution method. Serial dilutions were plated on MHA plates and cultivated in an incubator (37°C, 5% CO_2_) for 18 h for bacterial count determination. The untreated control mice (treated with 0.2 mL of 0.9% sterile physiological saline solution) were also sacrificed for the bacterial count estimation at 0 and 24 h.

The free cefquinome concentration in the murine thigh and in plasma for each dose regimens was extrapolated from the PK characteristics derived from the PK study described as above (such as from 0.04 and 0.01 mg/kg). After analysis using a non-compartmental model and combined with MIC, the PK/PD indices of *f* AUC_24h_/MIC, *f* C_max_/MIC, and %*f*T>MIC (the percentage of time the drug concentrations were above MIC over 24 h in the murine thigh) were calculated.

To evaluate the relationships between PK/PD parameters and antibacterial efficacy of cefquinome against *A. pleuropneumoniae*, an inhibitory sigmoid maximum effect (E_max_) model was used. The formula was described as follows: E = E_max_-(E_max_-E_0_) × ${\text{C}}_{\text{e}}^{\text{N}}$/(${\text{C}}_{\text{e}}^{\text{N}}$ + ${\text{EC}}_{50}^{\text{N}}$), where, E is the antibacterial effect, defined as the change of bacterial count in log_10_ (CFU/thigh) between before and at 24 h after drug administration. E_0_ is the maximum effect after administration of various drug dosages. E_max_ is the bacterial count change in the untreated control group. C_e_ is the PK/PD parameter. EC_50_ is the value of PK/PD surrogate of the drug producing 50% of the maximum antibacterial effect. N is the Hill coefficient that describes the slope of the PK/PD parameter-effect curve. The coefficients of determination (*R*^2^) were used to determine the relationships between PK/PD parameters and efficacy.

One-way ANOVA software (version 22, IBM) was used to analyze the statistic difference of results and *P* < 0.05 was considered to indicate significance. All results were determined by repeating the experiment thrice.

## Results

### MIC and RR of Microdialysis

The MIC of cefquinome against *A. pleuropneumoniae* was 0.0078 μg/mL in TSB, and this value was used for PK/PD integration. The *in vitro* RRs of microdialysis are listed in Table 1. The RRs determined by dialysis and retrodialysis decreased when the flow rate increased and were independent of cefquinome concentration. There was no statistical difference between RRs determined by dialysis and retrodialysis, which indicated that the retrodialysis method can be applied to detect *in vivo* recoveries. In the present study, the flow rate of 1.5 μL/min was used for subsequent experiments. The *in vivo* RRs fluctuated from 24.6 ± 7.5% to 43.3 ± 2.8%, with no statistical difference observed (*P* = 0.058) during 6 h. The mean value of *in vivo* RR was 0.373 which was used to calculate the free concentration of cefquinome in the murine thigh.

**Table 1:** *In vitro* relative recoveries of cefquinome in microdilysis probe determined by dialysis and retrodialysis in different perfusion rate and drug concentrations.

	**Relative recovery (%)**	
**Variables (unit)**	**Dialysis**	**Retrodialysis**
**Perfusion rate (μL/min)**		
0.5	54.7 ± 19.5	53.4 ± 17.0
1	38.6 ± 13.4	36.3 ± 7.1
1.5	24.0 ± 7.2	27.5 ± 4.4
2	19.1 ± 6.0	17.8 ± 5.6
**Concentration (ng/mL)**		
50	24.1 ± 0.8	23.5 ± 4.2
100	26.0 ± 0.9	24.6 ± 8.3
500	25.3 ± 2.0	25.9 ± 1.5

*Values are expressed as mean ± SD (Standard Deviation)*.

### Pharmacokinetics of Free Cefquinome in the Plasma and Thigh

The plasma protein binding levels of cefquinome ranged from 16.1 to 17.6% at various concentrations (5, 50, and 500 ng/mL), and the mean value was 16.8%. Therefore, 0.832 was applied to calculate the free concentrations of cefquinome in plasma.

**Figure 1:**
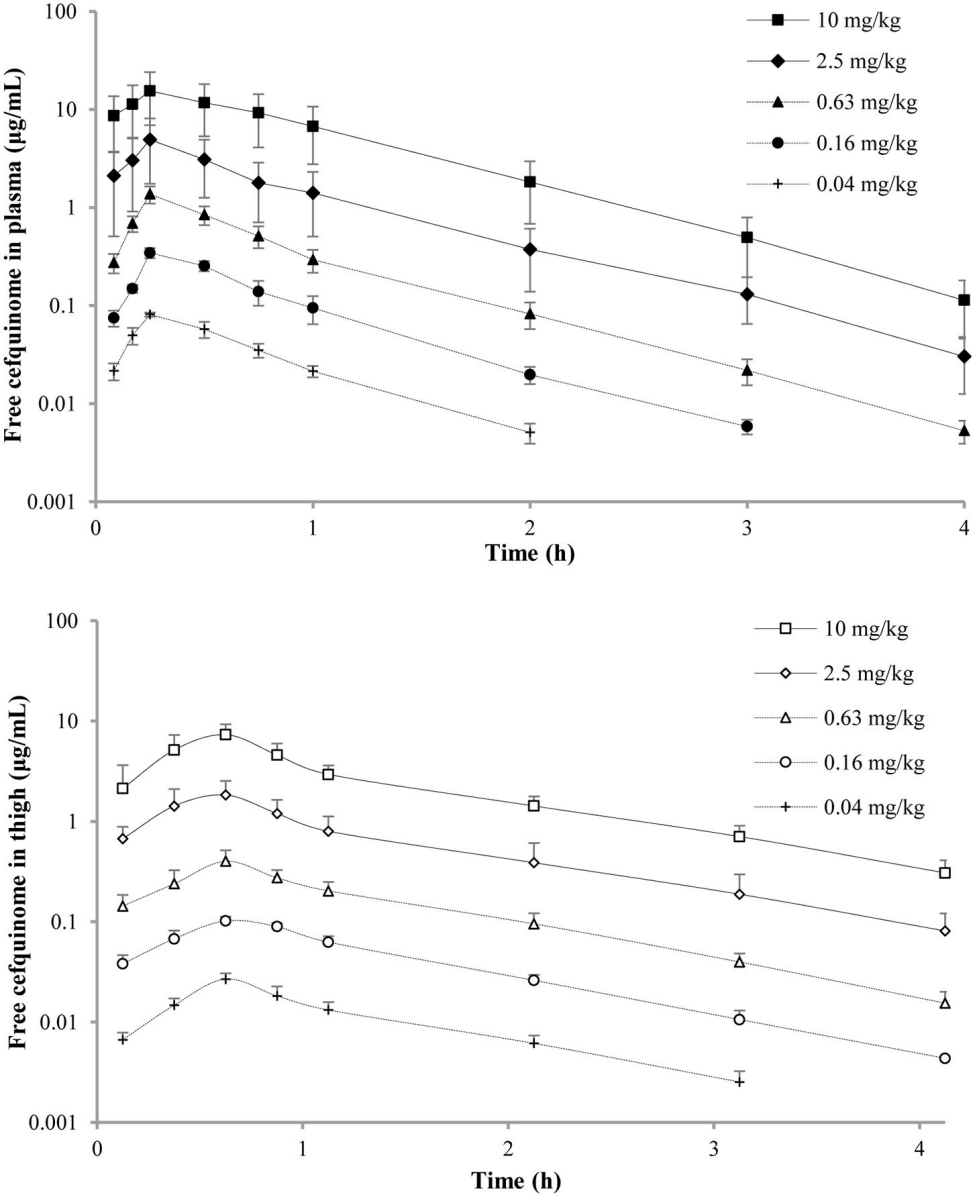
Pharmacokinetic curves of free cefquinome in plasma and thigh in the murine thigh infection model after administration of single doses of 0.04, 0.16, 0.63, 2.5, and 10 mg/kg by subcutaneous injection. Filled and open symbols represent concentrations in the plasma and thigh, respectively. Each symbol represents the mean ± SD of the values for eight mice.

The concentration-time curves of free cefquinome in plasma and thigh are depicted in Figure 1 and the PK indices are listed in Table 2. The AUC_inf_ (AUC from 0 h extrapolated to infinity) and C_max_ of cefquinome in plasma linearly increased when the dose escalated (AUC_last_, *R*^2^ = 0.989; C_max_, *R*^2^ = 0.988). Similar PK characteristics were observed in the thigh, but the values of AUC_inf_ and C_max_ were lower than those in plasma at the same dose. The T_1/2β_ of cefquinome in plasma and thigh ranged from 0.46 to 0.53 h and 0.77 to 0.96 h, respectively. The penetration of free cefquinome from plasma to thigh was determined by calculating the ratio of AUC_thigh_ to AUC_plasma_, and the values of penetration ranged from 0.56 to 0.63.

**Table 2:** Pharmacokinetic parameters of free cefquinome in plasma and thigh after a single subcutaneous dose in neutropenic mice with thigh infection.

	**Parameters (unit)**				
**Dose (mg/kg)**	**T_**1/2β**_ (h)**	**C_**max**_ (μg/mL)**	**AUC_**inf**_ (mg·h/L)**	**AUC_**inf**_/dose (kg·h/L)**	**AUC_**plasma**_/AUC_**thigh**_**
**Plasma**					
0.04	0.46 ± 0.06	0.08 ± 0.00	0.06 ± 0.01	1.55 ± 0.18	
0.16	0.50 ± 0.04	0.34 ± 0.01	0.26 ± 0.04	1.63 ± 0.24	
0.63	0.51 ± 0.02	1.37 ± 0.27	0.94 ± 0.16	1.49 ± 0.26	
2.5	0.53 ± 0.04	4.98 ± 3.11	3.87 ± 2.38	1.55 ± 0.95	
10	0.50 ± 0.06	15.48 ± 8.56	16.07 ± 8.94	1.61 ± 0.90	
Mean	0.50 ± 0.02	NA	NA	1.57 ± 0.05	
**Thigh**					
0.04	0.83 ± 0.12	0.03 ± 0.00	0.03 ± 0.00	0.87 ± 0.12	0.56
0.16	0.77 ± 0.04	0.10 ± 0.01	0.15 ± 0.02	0.97 ± 0.11	0.60
0.63	0.92 ± 0.07	0.40 ± 0.11	0.55 ± 0.13	0.88 ± 0.21	0.59
2.5	0.94 ± 0.14	1.83 ± 0.70	2.48 ± 1.04	1.00 ± 0.44	0.63
10	0.96 ± 0.10	7.32 ± 1.97	9.31 ± 2.34	1.00 ± 0.18	0.57
Mean	0.88 ± 0.07	NA	NA	0.94 ± 0.06	0.59 ± 0.02

*T_1/2β_, elimination half-life; AUC_inf_, area under the concentration-time curve from 0 h extrapolated to infinity; C_max_, maximum concentration of drug in in plasma or thigh dialysate. NA, not applicable. All the data were the mean value of 8 mice*.

### Relationships Between PK/PD Parameters and Antibacterial Efficacy

In untreated control mice, the initial bacterial population (2 h after infection) ranged from 6.23 to 6.53 log_10_ CFU/thigh and increased by 2.07 log_10_ CFU/thigh after 24 h.

**Figure 2:**
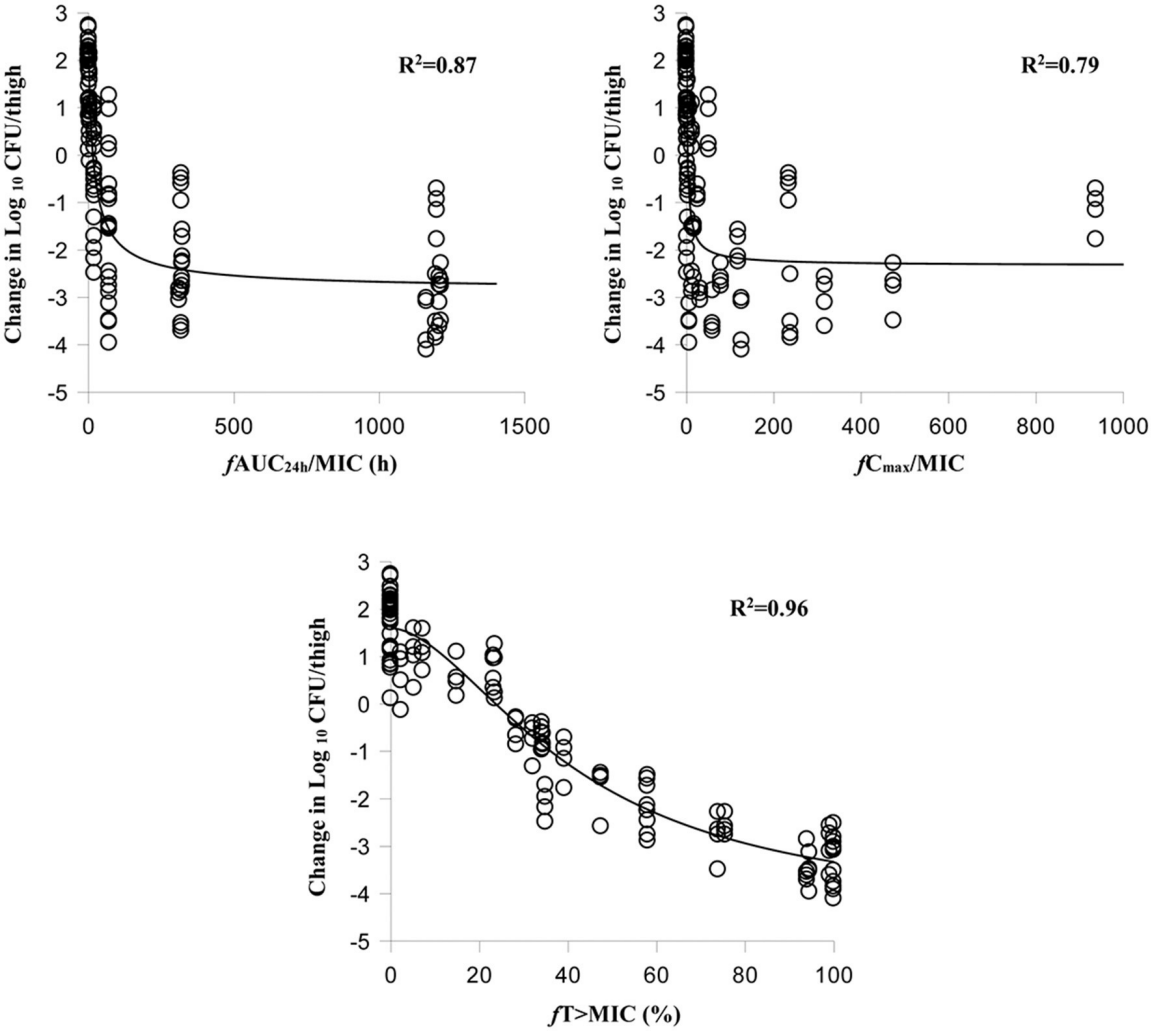
Relationships between antibacterial effect and three PK/PD parameters of free cefquinome in the thigh. %*f*T > MIC, the percentage of time that the free cefquinome concentration is above the MIC in the thigh. The *R*^2^ values are the coefficients of determination.

**Figure 3:**
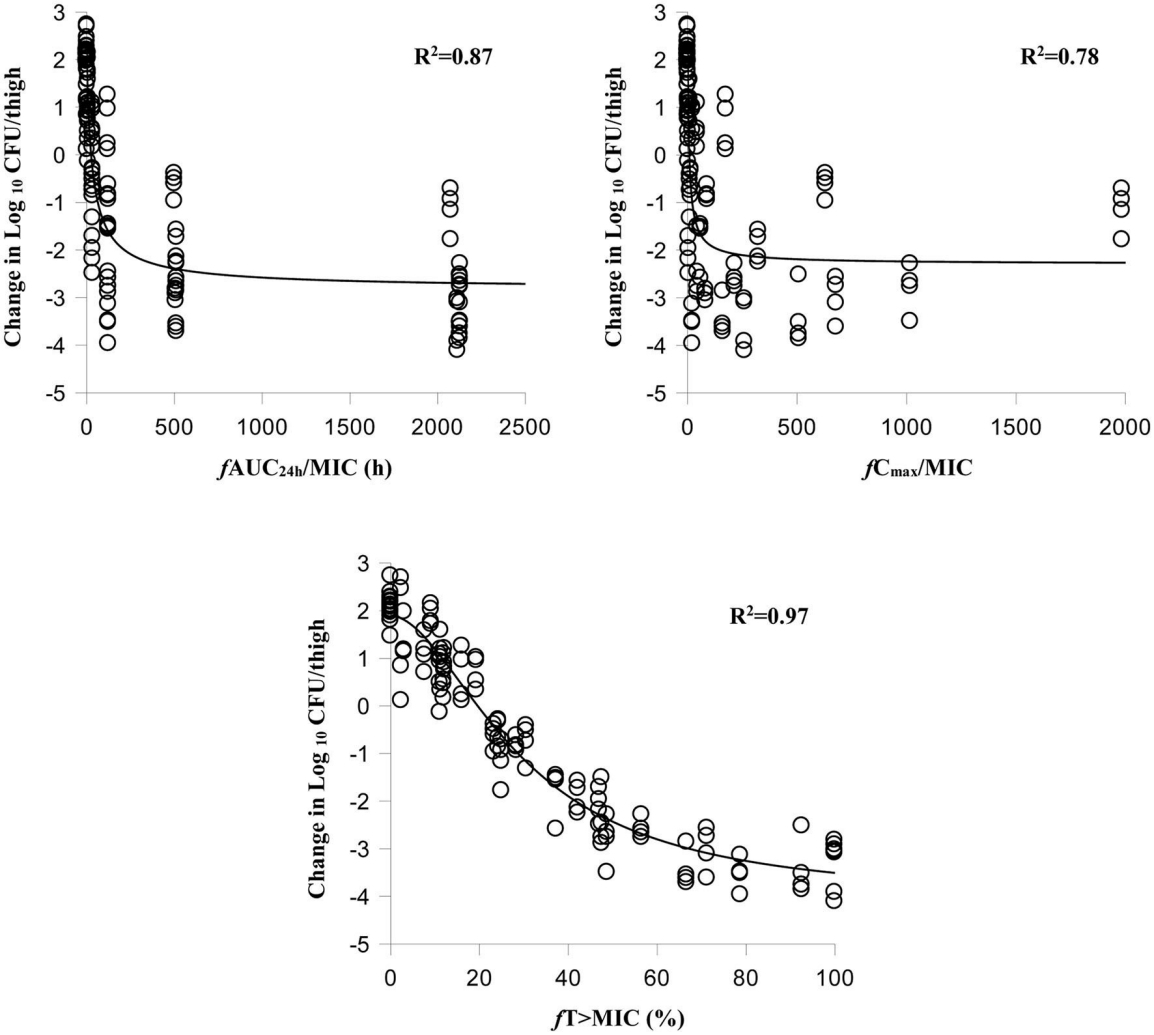
Relationships between antibacterial effect and three PK/PD parameters of free cefquinome in plasma. %*f*T > MIC, the percentage of time that the free cefquinome concentration is above the MIC in plasma. The *R*^2^ values are the coefficients of determination.

The relationships between antibacterial efficacy and PK/PD indices derived from the murine thigh and plasma are shown in Figures 2, 3, respectively. For murine thigh, the results shown that *f* %T > MIC (*R*^2^ = 0.96) was the fittest PK/PD index correlated to efficacy, in comparison with *f* C_max_/MIC (*R*^2^ = 0.79) and *f* AUC_24h_/MIC (*R*^2^ = 0.87). For plasma, the results also shown that *f* %T>MIC (*R*^2^ = 0.97) was the fittest PK/PD index correlated to efficacy compared to *f* C_max_/MIC (*R*^2^ = 0.78) and *f* AUC_24h_/MIC (*R*^2^ = 0.87). We also evaluated the values of %*f*T > MIC to achieve different antibacterial efficacy and the obtained values for E_max_, E_0_, EC_50_, and N are listed in Table 3. The estimated values of %*f*T > MIC for achieving a net static reduction, a 1-log_10_ CFU reduction, a 2-log_10_ CFU reduction, and a 3-log_10_ CFU reduction were 19.56, 28.65, 41.59, and 67.07% in plasma and 21.74, 36.11, 52.96, and 82.68% in murine thigh, respectively.

**Table 3:** The values of PK/PD parameters and %*f*T > MIC required to achieve various degrees of antibacterial efficacy in murine plasma and thigh.

**Parameters**	**Values in plasma**	**Values in thigh**
E_max_ (Δlog_10_ CFU/thigh)	1.89	1.60
E_0_ (Δlog_10_ CFU/thigh)	−4.08	−4.40
EC_50_ (%)	29.65	42.06
Slope (*N*)	1.85	1.76
%*f*T>MIC for net static effect in 24 h (%)	19.56	21.74
%*f*T>MIC for 1-log reduction in 24 h (%)	28.65	36.11
%*f*T>MIC for 2-log reduction in 24 h (%)	41.59	52.96
%*f*T>MIC for 3-log reduction in 24 h (%)	67.07	82.68

*%fT>MIC, the percentage of time that free drug concentration in murine plasma or thigh remains above the MIC. E_0_, the maximum effect after administered various cefquinome dosages. E_max_, the change of bacterial count in control group. EC_50_, the %fT > MIC values required to achieve 50% of the maximal antibacterial effect during 24 h. N, the Hill coefficient which describes the steepness of the %fT > MIC and effect curve*.

## Discussion

Porcine pleuropneumonia, caused by *A. pleuropneumoniae*, is a severe respiratory disease and is a serious threat to the pig breeding industry (2, 30). Based on the composition of capsular polysaccharide, there are 16 serovars of *A. pleuropneumoniae* have been found until now. And based on the requirement of NAD to grow, *A. pleuropneumoniae* can be classified as biovar I (NAD-dependent, typical) and biovar II (NAD-independent, atypical). The main virulence of *A. pleuropneumoniae* is Apx toxins (ApxI, II, III, and IV) which can produce cytotoxicity and haemolytic activity. The way of transmission was mainly by direct nasal contact or by aerosol spread (by coughing or sneezing). The predominant symptoms include anorexia, hyperpyrexia, and severe respiratory distress. The lung of infected pigs were gradually damaged and surrounded by fibrotic tissues. Common antibacterial drugs such as florfenicol, fluoroquinolones, and cephalosporins have been used to treat this disease. However, the pathogenic bacteria may develop resistance as a result of inappropriate use of the antibiotics (2, 31, 32). To reduce the possibility of resistance, PK/PD integration models have been frequently applied to optimize the dosage regimens. The neutropenic murine infection model is a PK/PD model that is often used to study the relationships between the PK parameters of drugs in plasma and their efficacy in infected tissues (15–17, 19–21). However, the plasma PK/PD parameters may not actually reflect the antibacterial efficacy in infected tissues. Therefore, assessments of the relationship between PK/PD parameters of antibiotics in the target tissue and their efficacy are both necessary and valuable. Microdialysis technology can resolve this problem and may be widely used in a number of pharmaceutical research studies in the future. *A. pleuropneumoniae* is a pathogen which can cause severe respiratory disease and can easily infect lung and tonsilla. Therefore, logically speaking, it is ideal to study the PK of cefquinome in pig lung infected with *A. pleuropneumoniae*. However, in our preliminary study, because the murine lung is fragile and small, the mouse were easy to die when we implanted microdialysis probe into the lung. Therefore, we finally selected a murine thigh infection model to study the cefquinome PK and assess the relationships between PK/PD parameters and efficacy. And murine thigh infection model can study the PK of cefquinome with little damage to mouse when implanted microdialysis probe into murine thigh (without surgery). Therefore, we considered that murine thigh infection model was a feasible and valuable method to study the relationships between PK/PD parameters and efficacy by microdialysis.

In the present study, we used a microdialysis technique to explore the pharmacokinetics of free cefquinome in the thigh and plasma of a neutropenic murine thigh infection model. The *in vitro* RRs of the probe were concentration-independent and decreased when the flow rate increased. There was no significant difference between the RRs determined by dialysis and retrodialysis. These results were consistent with the findings of other reports (24) and indicate that the microdialysis probe was suitable to study the pharmacokinetics of cefquinome by retrodialysis *in vivo*. The *in vivo* RRs were slightly higher than the *in vitro* RRs. One explanation is that the external condition of the microdialysis probe is different *in vitro* and *in vivo*. *In vitro*, the probe is encircled by Ringer's solution. However, *in vivo*, the probe is surrounded by cells, blood vessels, and interstitial fluid. When the RR was determined *in vivo*, cefquinome would penetrate from the microdialysis membrane to blood vessels and circulate in the body of mice and would be metabolized and eliminated by the metabolic system of mice. Therefore, the concentration of cefquinome in the thigh dialysate would be low, resulting in a high RR *in vivo*.

Cefquinome is time-dependent antibacterial drug that exerts its antibacterial effect based on the value of %T > MIC. This antibacterial characteristic has been proved by other studies (9–11, 14, 19–21) and was also observed in our study. After analysis using an inhibitory sigmoid E_max_ model, the %*f*T > MIC (*R*^2^ = 0.97) showed the best correlation to the efficacy compared to the *f* AUC/MIC (*R*^2^ = 0.87) and *f* C_max_/MIC (*R*^2^ = 0.79) in murine thigh. Meanwhile, a same results can be found in plasma. These results indicate that it is possible to estimate the antibacterial efficacy by applying the PK/PD parameters derived from the thigh.

The magnitudes of %*f*T > MIC to predict different antibacterial effects have been studied in other experiments. Wang et al. (20) used a neutropenic mouse thigh model to study the pharmacodynamics of cefquinome against *Staphylococcus aureus* and the magnitudes of %*f*T > MIC required to achieve net bacterial stasis, 0.5-log_10_ CFU reduction, and 1-log_10_ CFU reduction were 31.61, 38.48, and 54.01%, respectively. Another experiment (19) explored the activity of cefquinome against *Escherichia coli* in the thigh of neutropenic mice, and the magnitudes of %*f*T > MIC required to accomplish net bacterial stasis, 1-log_10_ CFU reduction, and 2-log_10_ CFU reduction were 28.01%, 37.23%, and 51.69%, respectively. However, all the above results obtained in other studies were derived from free cefquinome in plasma. In our study, we used the data obtained from murine thigh and plasma to evaluate the magnitudes of %*f*T > MIC required to achieve different antibacterial effects. The magnitudes of %*f*T > MIC in plasma to achieve static reduction, 1-log_10_ CFU reduction, 2-log_10_ CFU reduction, and 3-log_10_ CFU reduction were 19.56, 28.65, 41.59, and 67.07%, respectively. However, a bigger value of %*f*T>MIC was needed to achieve the same antibacterial efficacy based on thigh data (21.74, 36.11, 52.96, and 82.68%, respectively). There are several reasons may explain these phenomenon. One reason may be that the distribution of cefquinome in thigh was slower than in plasma. Therefore, cefquinome need a longer time to reach an antibacterial concentration in thigh compared to in plasma. Another reason was probably because the cells and interstitial space were destroyed by bacteria. In general, the PK characteristics of antibacterial agents differ in elimination half-life, concentration, and protein binding between in plasma and in tissue. Moreover, the PK characteristics of drugs in tissues are more easily affected by a bacterial infection, which can destroy the cells and interstitial fluid of organs. Therefore, in studies on the PK/PD integration of antibacterial agents against pathogens, it is more reasonable to use the PK parameters derived from infected tissues than plasma. Hence, our results will provide some valuable references for using microdialysis to explore the PK/PD integration of drugs against bacteria in infected tissues.

Although we could successfully evaluate the relationships between PK/PD parameters and antibacterial efficacy in the murine thigh by the microdialysis method, our experiment had several deficiencies. One problem is that the PK data of cefquinome in the murine thigh over 24 h was obtained by the extrapolative method. However, the extrapolated PK data were slightly different from the actual PK characteristics of cefquinome in murine thigh infected with bacteria. Because the cells and interstitial space in the thigh tissue were destroyed over time after bacterial infection (33), the penetration of cefquinome from blood to tissue probably decreased, and the PK characteristics may no longer be regular in the murine thigh, especially in the low-dose groups. In our present study, because the lung was fragile and lung microdialysis was difficult in mice, we chose a murine thigh infection model to evaluate the application of microdialysis in PK/PD integration. However, the infectious target sites of *A. pleuropneumoniae* are in the respiratory system in pigs, in regions such as the lung, trachea, and pleura. Therefore, the PK characteristics of cefquinome would show some differences between the thigh and lung. In this experiment, we primarily used microdialysis to assess the PK/PD integration, and more detailed studies should be performed to explore the application of microdialysis in PK/PD integration, e.g., studies with a longer sampling time (24 h after drug administration) and a different target organ infection model (lung infection model).

In conclusion, the present experiment successfully studied the PK properties of cefquinome in the thigh and plasma in a murine thigh infection model by microdialysis. After the relationships between PK/PD parameters and efficacy were analyzed, the %*f*T > MIC was found to be the fittest PK/PD parameter to predict the antibacterial effect. The PK/PD parameters obtained from the thigh could successfully predict the antibacterial effect and actually reflect the antibacterial effect. The %*f*T > MIC values required to achieve a bactericidal effect in plasma and thigh were 67.07 and 82.68%, respectively. These results indicated that microdialysis is suitable and valuable for exploring PK/PD integration of drugs against pathogens. These data will also provide some basis for designing cefquinome dosages to treat respiratory tract disease caused by *A. pleuropneumoniae*.

## Data Availability Statement

All datasets presented in this study are included in the article/supplementary material.

## Ethics Statement

The animal study was reviewed and approved by Committee on the Ethics of Animals of South China Agricultural University.

## Author Contributions

HD and LZ: conceived and designed the experiments. LZ and ZZ: performed the experiments. LZ: analyzed data and drafted the article. XG, SH, XS, and HD: contributed to the revision. All authors: read and approved the final manuscript.

## Conflict of Interest

The authors declare that the research was conducted in the absence of any commercial or financial relationships that could be construed as a potential conflict of interest.
